# Genotypic diversity, antimicrobial resistance and biofilm-forming abilities of *Campylobacter* isolated from chicken in Central China

**DOI:** 10.1186/s13099-017-0209-6

**Published:** 2017-11-09

**Authors:** Tengfei Zhang, Jun Dong, Yiluo Cheng, Qin Lu, Qingping Luo, Guoyuan Wen, Guoping Liu, Huabin Shao

**Affiliations:** 1 Key Laboratory of Prevention and Control Agents for Animal Bacteriosis, Institute of Animal Husbandry and Veterinary, Hubei Academy of Agricultural Sciences, Wuhan, China; 2 College of Animal Science, Yangtze University, Jingzhou, China; 3 Hubei Key Laboratory of Animal Embryo and Molecular Breeding, Institute of Animal and Veterinary Science, Hubei Academy of Agricultural Sciences, Wuhan, China; 4 The Cooperative Innovation Center for Sustainable Pig Production, Wuhan, China

**Keywords:** *Campylobacter*, Prevalence, Antibiotic resistance, Genotype lineage, Biofilm

## Abstract

**Background:**

*Campylobacter* is considered to be the leading cause of human bacterial gastroenteritis, of which poultry is the main reservoir. *Campylobacter* contaminated chicken products are a major cause of human *Campylobacter* infection. In this study, the prevalence of *Campylobacter* in chicken in central China was investigated, and the genotypic diversity, antimicrobial resistance and biofilm of these isolates were characterized.

**Results:**

A total of 206 *Campylobacter* isolates, including 166 *C. jejuni* and 40 *C. coli*, were isolated from chicken farms and live poultry markets in central China. Multilocus sequence typing and phylogenetic analysis showed that the *Campylobacter* isolates had diverse genetic backgrounds, which covered most of the dominant clone complexes (CCs) reported throughout China. The most prevalent CCs were CC-464, CC-1150, CC-353, and CC-828. All the isolates showed resistance to norfloxacin, ciprofloxacin and Cefazolin, and a prevalent resistance to fluoroquinolones, β-lactams and tetracyclines was also observed. Among all the isolates, 133 strains showed the ability to form biofilm, thereinto, the isolates in two genetic branches, mainly including CC-21, CC-48, CC-677 and CC-45, showed a significantly lower ability to form biofilm than other genetic branches (*p* < 0.05). However, in general, the ability to form biofilm varied among different genetic branches, suggesting a complex genetic background to biofilm formation, but not only the genetic lineages. Compared with the strains unable to form biofilm, biofilm-producing strains possessed a significantly higher resistance to ampicillin, neomycin, sulfamethoxazole, amikacin, clindamycin and erythromycin (*p* < 0.05).

**Conclusions:**

To the best of our knowledge, this is the first report on the relationship of the genotypic diversity, antimicrobial resistance and biofilm-forming abilities of *Campylobacter* isolated from chicken in Central China, which showed the potential importance of biofilm in antimicrobial resistance. This study will help us better understand the epidemiology and antimicrobial resistance of *Campylobacter*.

**Electronic supplementary material:**

The online version of this article (10.1186/s13099-017-0209-6) contains supplementary material, which is available to authorized users.

## Background

*Campylobacter* is considered to be the leading cause of human bacterial gastroenteritis worldwide [1], accounting for an estimated 500 million infections per year globally [2]. In severe cases of *C. jejuni* infection, individuals may develop post infection complications associated with Guillain Barré Syndrome [3]. In North China, 36 cases of Guillain Barré Syndrome, resulted from *C. jejuni* infection, were reported in 2007 [4].

*Campylobacter* species, mainly including *C. jejuni* and *C. coli*, widely colonize in the intestinal tract of wild and domesticated animals and birds [5–7]. Chicken is one of the most popular animal-based food sources worldwide, which is also the reservoir of *Campylobacter*. *Campylobacter*-contaminated chicken products are a major cause of human *Campylobacter* infection [8], which highlights its potential public health threat. Several epidemiologic studies on *Campylobacter* have been carried out in some parts of China. From 2008 to 2014, Wang et al. reported that the positive rates of *C. jejuni* and *C. coli* were 18.1 and 19.0% respectively in five provinces of China [9]. Zhang et al. analyzed the genetic diversity of the *C. jejuni* isolates in Eastern China by multilocus sequence typing (MLST) and identified 94 sequence types (STs) belonging to 18 clonal complexes (CCs) [10]. However, data on the prevalence and genetic diversity of *Campylobacter* is still limited in China, especially central China, which is an important transportation junction.

Moreover, *Campylobacter* isolates have raised great concerns due to a frequent emergence of resistance to fluoroquinolone, erythromycin, and other drugs [11, 12], which limits treatment alternatives. Therefore, analysis of antimicrobial resistance of *Campylobacter* in the poultry industry will contribute to managing cognate infections and mitigating the emergence of antimicrobial resistant strains. Recent years, the multidrug-resistant *Campylobacter* have been frequently isolated, and a high antimicrobial resistance rate of *Campylobacter*, especially to fluoroquinolone, has been reported in many areas [13–15]. Multi-drug resistance of *Campylobacter* is more severe in China where the resistance to fluoroquinolones was reported to be as high as 98% in some areas [16, 17]. Although some of the mechanisms accounting for antimicrobial resistance in *Campylobacter* have been revealed [11, 18, 19], some possible factors may also attribute to the raise of antimicrobial resistance, such as the ability of biofilm formation.

Our previous study has shown that the *Campylobacter* positive rate was 17.2%, with bacterial count varying from 3.6 to 360 most-probable-number (MPN)/g in the positive samples of chicken meats collected from markets in central China [20]. Studying the prevalence of *Campylobacter* in live chicken and their surroundings will help us further control these pathogens. In this study, we investigated the prevalence, antimicrobial resistance and genetic diversity of *Campylobacter* strains isolated from chicken farms and markets in central China, which is one of the most important livestock and poultry circulation centers. We also tested the biofilm-forming ability of the *Campylobacter* isolates and analyzed the potential correlation among biofilm formation, genotypes, and antimicrobial resistance.

## Methods

### Sampling and isolation of *Campylobacter*

From 2012 to 2016, a total of 817 samples, including 317 anal swabs, 15 soils and 12 aerosols collected from chicken farms, and 448 anal swabs, 15 soils and 10 aerosols collected from live poultry markets, were collected in central China (3 farms and 4 markets in Hubei, 2 farms and 3 markets in Henan, 2 farms and 2 markets in Jiangxi and 1 farm and 2 markets in Anhui). In each sampling site, 35–45 anal swabs were collected. In parts of sampling sites, 2 aerosol samples and 3 soil samples were collected. Freshly collected anal swabs or soils were kept into Cary-Blair modified transport media (AMRESCO, USA). Aerosols (375 L/sample) were collected using BioSamper (SKC Ltd, USA). The samples were transported to the laboratory for *Campylobacter* isolation. The samples were resuspended in PBS which were used to inoculate Bolton broth containing *Campylobacter* growth and selective supplements (Oxoid, England) and incubated at 42 °C for 24 h in air tight jars containing the AnaeroPack (Mitsubishi, Japan) to generate a microaerobic condition. 100 µl of the culture was spread onto a modified charcoal cefoperazone deoxycholate agar (mCCDA, Oxoid) plate containing *Campylobacter* selective supplements and incubated for 48 h at 42 °C under microaerobic condition [21]. The suspected *Campylobacter* colonies were further purified and identified by PCR as described [22]. *C. jejuni* and *C. coli* were differentiated by hippuric acid hydrolysis test and PCR test [6]. The identified *Campylobacter* strains were stored at − 80 °C in MH broth containing 30% (v/v) glycerol.

### Antibiotic resistance profiles

The antibiotic susceptibility of the isolates was determined by the disk diffusion method on Mueller-Hinton Agar (MHA, Oxoid) according to the Clinical and Laboratory Standards Institute Standards guidelines [23]. A total of 11 antibiotics were tested, including ampicillin (Amp, 10 μg), Ceftriaxone (Cet, 30 μg), Cefazolin (Cez, 30 μg), amikacin (Ami, 30 μg), Neomycin (Neo, 30 μg), tetracycline (Tet, 10 μg), sulfamethoxazole (Sul, 300 μg), clindamycin (Cli, 10 μg), erythromycin (Ery, 10 μg), ciprofloxacin (Cip, 5 μg) and norfloxacin (Nor, 10 μg). After incubation for 24 h at 37 °C, the diameters (in mm) of the inhibition zones were measured. *E. coli* strain ATCC 25922 and *C. jejuni* strain ATCC 33560 were used as the quality control.

### Biofilm assays

Biofilm formation was assessed as described [24]. Briefly, overnight cultured cells were adjusted to an OD_590nm_ of 0.1 in Brucella medium (Oxoid) supplemented with 5% (v/v) chicken juice. To allow biofilm formation, 1 ml of the cell culture was added to a 24-well polystyrene tissue culture plate (Corning) which was incubated at 37 °C under microaerobic condition for 48 h before staining. For crystal violet staining, cells were discarded and each well was washed with water, and the plate was then dried at 60 °C for 30 min. One milliliter of 1% crystal violet solution was added to each well, and the plate was incubated on a rocker at room temperature for 30 min. Unbound crystal violet was washed off with water and the plate was dried at 37 °C. Bound crystal violet was dissolved in 20% (v/v) acetone-containing ethanol for 10 min. The dissolved crystal violet was then poured into cuvettes and OD_630nm_ was measured. All the tests were performed in triplicate. Three wells were subject to the same treatment but without bacteria inoculated, which were used as the negative control. The cutoff OD value (ODc) was defined as two times of the negative control value as previously reported [25, 26]. Based on the OD values, strains were classified into the following three categories: non-biofilm producer (OD ≤ ODc), weak biofilm producer (ODc < OD ≤ 2 × ODc) and strong biofilm producer (2 × ODc < OD).

### MLST typing

In order to determine the genetic diversity of the *Campylobacter* isolates and their relationship, MLST analysis was carried out as previously described [27]. Briefly, genomic DNA was extracted using MiniBEST Universal Genomic DNA Extraction Kit (TaKaRa, Dalian, China) according to the manufacturer’s instructions. MLST analysis was conducted by sequencing seven *Campylobacter* housekeeping genes (*aspA*, *glnA*, *gltA*, *glyA*, *pgm*, *tkt*, and *uncA*). The primers sets for these seven genes and their amplification conditions were used as previously described [27]. Amplification products were purified and sequenced. Allele numbers, sequence types (STs) and clonal complexes (CCs) were assigned using the *Campylobacter* MLST database (http://pubmlst.org/Campylobacter/). Novel STs were submitted to MLST database and assigned new numbers. Consensus network of the calculated tree was constructed by SliptsTree 4 version 1.2 using the multi-aligned core genome sequence of the different STs.

### Statistical analysis

Chi square was used to determine the significance of resistance rates in different biofilm-forming groups and to compare the isolation rates of *C. jejuni* and *C. coli*. For analyzing the biofilm-forming abilities, the mean OD values (mean ± SEM) of isolates in each clade were calculated, and two-tailed t tests were used to determine the significance of the biofilm-forming abilities in different clades *p* < 0.05 was considered statistically significant.

## Results

### Prevalence of *Campylobacter*

As shown in Table 1, 206 *Campylobacter* strains (positive rate 25.2%) were isolated, including 156 *C. jejuni* and 38 *C. coli* from anal swab samples, 5 *C. jejuni* and 2 *C. coli* from soil samples, and 5 *C. jejuni* from aerosol samples. The isolation rate of *C. jejuni* was higher than that of *C. coli* (20.3% vs 4.9%, *p* = 0.000). Among these isolates, 66 *C. jejuni* (positive rate 19.2%) and 19 *C. coli* (positive rate 5.5%) were isolated from the 344 samples collected from poultry farms, and 100 *C. jejuni* (positive rate 21.1%) and 21 *C. coli* (positive rate 4.4%) were isolated from the 473 samples collected from live poultry markets.

**Table 1:** The prevalence of *Campylobacter* in chicken farms and poultry markets in central China

Sources	Sampling site (no.)	No. of positive samples/collected samples							
		Anal swabs		Soils		Aerosols		Total	
		*C. jejuni*	*C. coli*	*C. jejuni*	*C. coli*	*C. jejuni*	*C. coli*	*C. jejuni*	*C. coli*
Chicken farms	Hubei (3)	23/112	8/112	1/9	0/9	4/8	0/8	66/344	19/344
	Henan (2)	12/83	5/83	1/6	1/6	1/4	0/4		
	Anhui (2)	15/82	4/82	N/A	N/A	N/A	N/A		
	Jiangxi (1)	9/40	1/40	N/A	N/A	N/A	N/A		
Poultry markets	Hubei (4)	32/167	9/167	1/9	1/9	0/6	0/6	100/473	21/473
	Henan (3)	31/117	4/117	2/6	0/6	0/4	0/4		
	Anhui (2)	19/86	4/86	N/A	N/A	N/A	N/A		
	Jiangxi (2)	15/78	3/78	N/A	N/A	N/A	N/A		
Total		156/765	38/765	5/30	2/30	5/22	0/22	166/817	40/817

### Genetic diversity of *Campylobacter*

**Fig. 1:**
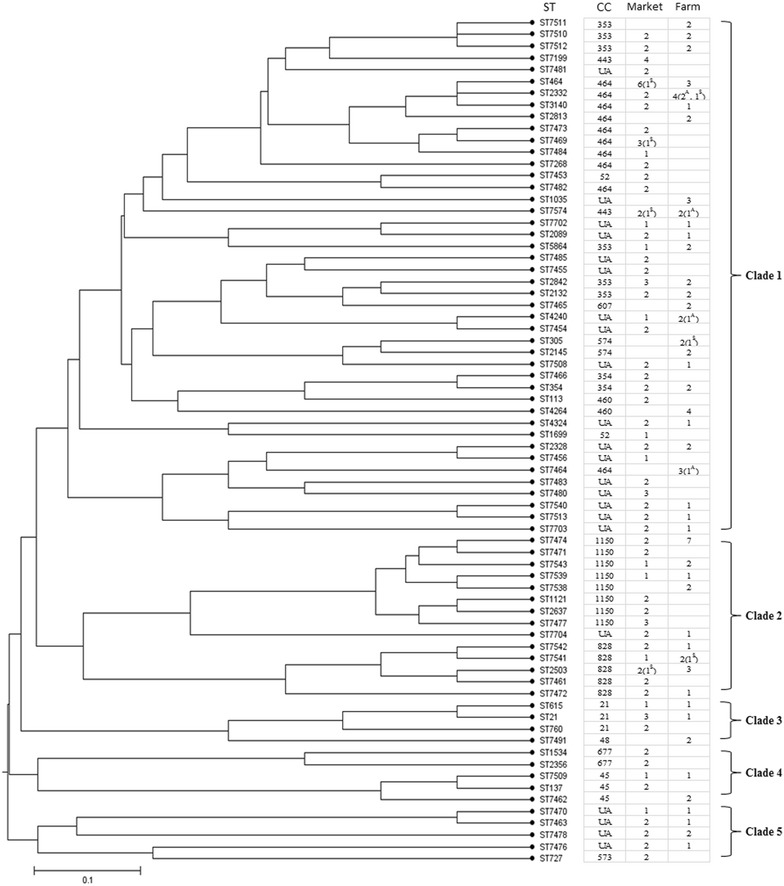
The genetic relationships of all STs in this study. All the STs were clustered to five major clades. The CCs and the numbers of strains isolated from chicken farms and poultry markets respectively in each ST were also listed. The numbers of strains isolated from soils (marked with “S”) or aerosols (marked with “A”) were listed in bracket

MLST was performed to determine the genetic diversity and clonal origins of the *Campylobacter* isolates, and the details of MLST results have been listed in Additional file 1: Table S1. As shown in Fig. 1, 206 isolates contained a total of 72 different STs in our test. Among these isolates, 146 out of the 206 isolates possessed 50 different STs which belonged to 15 CCs. The remaining 60 isolates belonged to 22 different unassigned STs. 40 novel STs harboring 106 isolates were isolated for the first time. CC-464 was the most frequently isolated clonal complex which contained 33 isolates belonging to ten STs, and accounted for 16.0% (33/206) of all the isolates. The major clonal complexes also included CC-1150 (n = 25, 12.1%), CC-353 (n = 22, 10.7%) and CC-828 (n = 16, 7.8%). The isolates collected from markets covered 61 STs belonging to 12 CCs and unassigned, while those isolated from chicken farms harbored 45 STs belonging to 12 CCs and unassigned.

All the STs were classified into five major clades (Fig. 1). Clade 1 covered most of the isolates, including the two major clonal complexes CC-464 and CC-353. All of the *C. coli* isolates belonging to CC-1150 and CC-828 were clustered in clade 2. The isolates recovered from chicken markets and farms shared nine out of the fifteen clonal complexes and all the clades contained isolates recovered from chicken farms and markets, suggesting that the isolates collected from the two places might share same origins.

### Antimicrobial resistance profiles of *Campylobacter*

**Fig. 2:**
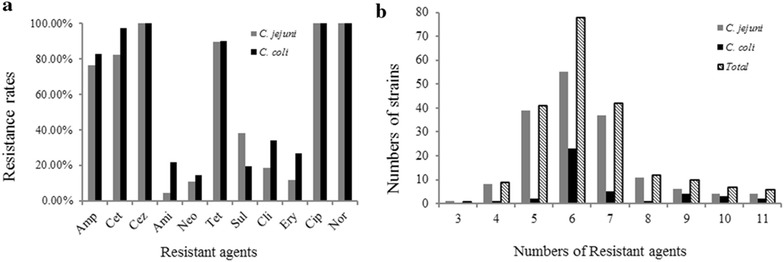
Antimicrobial resistance of *Campylobacter* isolates. **a** Resistance rates of *C. jejuni* and *C. coli* isolates to 11 agents; **b** multidrug resistance of *C. jejuni* and *C. coli* isolates to 11 agents

As shown in Fig. 2a, all of the *C. jejuni* and *C. coli* isolates showed resistance to norfloxacin, ciprofloxacin and Cefazolin. A high rate of resistance to tetracycline, ceftriaxone and ampicillin has also been observed for the isolates, among which 89.69% of the *C. jejuni* and 90.24% of the *C. coli* were resistant to tetracycline; 82.42% of the *C. jejuni* and 97.56% of the *C. coli* were resistant to ceftriaxone; and 76.36.5% of the *C. jejuni* and 82.93% of the *C. coli* were resistant to ampicillin. The isolates showed a relative low rate of resistance to amikacin (4.85% of the *C. jejuni* and 21.95% of the *C. coli*), neomycin (10.91% of the *C. jejuni* and 14.63% of the *C. coli*), and erythromycin (12.12% of the *C. jejuni* and 26.83% of the *C. coli*). Except for sulfamethoxazole and the three drugs which all the isolates were resistant to, the resistance rates of *C. jejuni* were lower than that of *C. coli* in this study.

As shown in Fig. 2b, all the isolates were resistant to at least three tested antimicrobial agents, among which 95.1% of the isolates were resistant to more than five antibiotics. There were four *C. jejuni* and two *C. coli* strains that were resistant to all the eleven antimicrobial agents tested. However, one C*. jejuni* strain showed sensitivity to most of the antibiotics, which was only resistant to three of antimicrobial agents tested. In general, most of the isolates were resistant to 5–7 antimicrobial agents tested. The most frequent multidrug resistance pattern was resistance to ciprofloxacin, norfloxacin, tetracycline, ampicillin, ceftriaxone and cefazolin, which covered 54 isolates (Additional file 1: Table S1).

### Biofilm formation of *Campylobacter* isolates

**Fig. 3:**
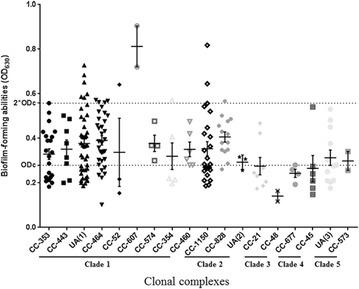
Biofilm-forming abilities of *Campylobacter* isolates belonging to different clonal complexes. The bottom broken lines indicate the cutoff value (ODc = 0.279) and twofold cutoff value (ODc = 0.558). Based on the OD values, the strains were classified in three categories: non-biofilm producer (OD ≤ ODc), weak biofilm producer (ODc < OD ≤ 2 × ODc) and strong biofilm producer (2 × ODc < OD)

The cutoff OD value (ODc) to define a biofilm producer was determined as OD_630nm_ = 0.279 as previously described [26]. The OD_630nm_ values generated by crystal violet staining of each isolate were listed in Additional file 1: Table S1. Based on the biofilm-forming ability, 206 *Campylobacter* isolates were classified to three groups (Table 2, Fig. 3). Seventy-three isolates (35.4%) were identified as non-biofilm producers (OD_630_ ≤ 0.279), while 133 isolates (64.6%) were biofilm producers. Among these biofilm producers, 113 isolates were weak biofilm producers (0.279 < OD_630_ ≤ 0.558) and 20 isolates were strong biofilm producers (OD_630_ > 0.558). All the soil isolates and aerosol isolates were biofilm producers.

**Table 2:** Antimicrobial resistance of *Campylobacter* isolates with different biofilm-forming abilities

Classes	Members	Biofilm strong isolates (n = 20)		Biofilm weak isolates (n = 113)		Biofilm negative isolates (n = 73)	
		No. of resistant isolates	Resistance rates (%)	No. of resistant isolates	Resistance rates (%)	No. of resistant isolates	Resistance rates (%)
β-lactams	Ampicillin	19	100	98	85.8	43	58.9
	Ceftriaxone	19	95.0	97	85.8	60	82.2
	Cefazolin	20	100	113	100.0	73	100.0
Aminoglycosides	Neomycin	11	55.0	13	11.5	2	2.7
	Amikacin	8	40.0	9	8.0	0	0
Tetracyclines	Tetracycline	16	80.0	99	84.1	70	95.9
Sulfonamides	Sulfamethoxazole	19	95.0	46	40.7	6	8.2
Fluoroquinones	Ciprofloxacin	20	100	113	100.0	73	100.0
	Norfloxacin	20	100	113	100.0	73	100.0
Lincosamides	Clindamycin	19	95.0	26	23.0	0	0
Macrolides	Erythromycin	18	90.0	13	11.5	0	0

### Correlation between biofilm formation and antimicrobial resistance

As shown in Table 2, both biofilm producers and non-biofilm producers showed a high rate of resistance to ceftriaxone, cefazolin, tetracycline and two fluoroquinolones drugs including ciprofloxacin and norfloxacin. However, compared with non-biofilm producers, biofilm producers possessed a higher rate of resistance to ampicillin (88.0% vs 58.9%, *p* = 0.000), neomycin (18.0% vs 2.7%, *p* = 0.002) and sulfamethoxazole (48.9% vs 8.2%, *p* = 0.000). Moreover, all of the non-biofilm producers were sensitive to amikacin, clindamycin and erythromycin. In contrast, strong biofilm producers showed a resistance rate of more than 90% to clindamycin and erythromycin. Six isolates, which were resistant to all the types of antibiotics tested, were biofilm producers. Among them, four out of the six isolates were strong biofilm producers (Additional file 1: Table S1). These results suggested that the ability of biofilm formation had a positive correlation with antimicrobial resistance of the *Campylobacter* isolates. However, there was an exception that compared with the non-biofilm producers, the biofilm producers showed a lower rate of resistance to tetracycline.

### Correlation between biofilm formation and genotypes

As shown in Figs. 1 and 3, 206 *Campylobacter* isolates fell into five clades, of which clade 1 and 2 had more members than the other clades. The mean OD values in each clade were as follows: clade 1 = 0.37 ± 0.01, clade 2 = 0.37 ± 0.02, clade 3 = 0.25 ± 0.04, clade 4 = 0.26 ± 0.03 and clade 5 = 0.31 ± 0.03. Although all of the strong biofilm producers were present in clade 1 and clade 2, strains in these two clades exhibited different levels of abilities to form biofilm, which suggested that the ability of biofilm formation varied among the dominant genotypes of *Campylobacter*. 75% of the strains (20/25) in clade 3 and clade 4 were non-biofilm producers and the ability of biofilm-formation of the strains in these two clades was significantly lower than that of the other clades (clade 3 vs clade 1, *p* = 0.007; clade 3 vs clade 2, *p* = 0.013; clade 4 vs clade 1, *p* = 0.012; clade 4 vs clade 2, *p* = 0.020). Strains which belonged to CC-21, CC-48, CC-677, CC-45 and a few unassigned isolates were included in these two clades.

## Discussion

Poultry are recognized as a main reservoir of *Campylobacter*. Consumption of poultry is considered to be an important cause of human infection with *Campylobacter*, and leads to extensive spread antimicrobial resistance [28]. In this study, *Campylobacter* strains were isolated from 25.2% of the samples collected from chicken farms and markets, including 166 *C. jejuni* and 40 *C. coli*. According to several previous reports, the positive detection rate of *Campylobacter* in poultry farms varies largely between different regions, ranging from 2 to 100%, and the prevalence of *Campylobacter* is lower in Scandinavian countries than in other European countries, North America, and developing countries [29]. China is the biggest developing country in which a diverse prevalence rate has also been reported in different parts of the country. For example, Huang et al. revealed that *C. jejuni* was frequently detected in poultry, with an average isolation rate of up to 18.61% [30]. Wang et al. showed that the positive rates of *C. jejuni* and *C. coli* were 18.1 and 19.0% respectively in chicken in five provinces of China [9]. In Tianjin, the contamination rates of *C. jejuni* and other *Campylobacter* species were 13.7 and 5.7% respectively [31]. In this study, our data showed that the positive rate of *C. jejuni* was a bit higher than most of the other studies carried out in China. We also found that *Campylobacter* existed in the soils and aerosols of chicken farms and markets, suggesting that the pathogens were widely spreading between host and surroundings. This situation makes it harder for us to control *Campylobacter* infection. A prevalence and risk assessment of *C. jejuni* in chicken in China suggested that key efforts should be made, especially in chick breeding and chicken preparation processes [32].

In our study, MLST analysis showed a total of 72 different STs belonging to 15 CCs and some unassigned clonal complexes. The major clonal complexes included CC-464, CC-1150, CC-353, and CC-828, which were similar to our previous investigation on chicken meat in the same region [21]. Most of these CCs (CC-464, CC-1150, CC-353 and CC-828) were also frequently identified in diarrhea patients worldwide [33, 34]. In North China, the most frequently isolated clonal complexes were CC-21, CC-353, CC-354 and CC-443 [31, 35], while the dominant clonal complexes of *C. coli* were CC-828 and CC-1150 [36]. In East China, the most common ST type of the *Campylobacter* strains isolated from human and food was ST-353, while the dominant ST type from chicken and food was ST-354 [10]. In Guangdong, a province in southern China, the dominant clonal complex was CC-828 [37]. It seems that the dominant clonal complexes of *Campylobacter* were discrepant in different regions. However, most of the CCs reported in these regions had been isolated in our study, which may be because central China, where all the samples were collected, is one of the most important livestock and poultry circulation centers in our country.

A total of 40 novel STs were identified in this study. Genetic relationship analysis showed that different sources of isolates have a crossed distribution in each clonal group and most of the novel STs only have a minor variation with a close phylogenetic relationship to known CCs. Selection forces, such as differences in temperature, structure and biochemical and immunological habitats, may accelerate the evolution to gain the ability to persist in different enteric environments and survive in different environments during transmission. Clade 1 and 2 contained lots of small genetic branches, which may be due to the adaptive evolution of isolates in these two clades occurred more frequently in our investigated regions.

Another more important selection pressure might be the usage of antibiotics, which could cause heritable genetic mutations and horizontal resistance gene transfer, leading to serious antimicrobial resistance in *Campylobacter* [11, 38]. More seriously, some of the antibiotics to which the *Campylobacter* isolates were resistant were used as therapeutic drugs in severe cases of infection [39]. Although resistance rates varied in different regions, in general high resistance rates, especially to fluoroquinolones, were found in most of the studies in China. For example, in Zhang et al.’s study, the resistance rate of *Campylobacter* to ciprofloxacin was 100%, and 94% to tetracycline, 61% to erythromycin, and 50% to ampicillin [36]. Chen et al. reported that more than 98% of the tested *Campylobacter* isolates were resistant to quinolones and tetracycline [40]. Even as early as in 2002, the prevalence of quinolone resistance of the isolates had been up to 85.9% in Hong Kong [41]. Low resistance rates of *Campylobacter* were only reported in Northwest China [42]. In our study, a very high resistance rate to β-lactams, tetracyclines and fluoroquinones was observed (Fig. 2), and a high resistance rate to the other drugs, such as erythromycin, was also found in the strong biofilm producers (Table 2). Our previous study showed that all of the fluoroquinolone-resistant strains contained a Thr-86-Ile substitution in GyrA, and that the CmeR-Box variations increased the expression of CmeABC efflux pump which led to the high resistance [43]. Overexpression of drug efflux pump may not only contribute to fluoroquinolones resistance, but also increase resistance to other drugs [44–46]. Bacteria exposing in efflux inhibitors or mutants in efflux pumps showed decreased biofilm, which suggested that efflux pumps also contributed to their biofilm formation [47, 48]. Although more resistance mechanisms need to be revealed, efflux pumps seem to play important roles in antimicrobial resistance as well as biofilm formation.

Biofilms are sessile communities of bacterial cells enclosed in a self-produced extracellular polysaccharide matrix, which plays an important role in evading host immune clearance and resisting antimicrobial agents, leading to persistent and chronic infections [26]. *Campylobacter* may form a monospecies biofilm, which protects them from environmental stress, including antibiotic treatment [24]. In our tested strains, 64.6% were identified to be biofilm producers. Comparing with the non-biofilm producers, the biofilm producers possessed a higher resistance rate to ampicillin, neomycin, sulfamethoxazole, amikacin, clindamycin and erythromycin. Although studies on the correlation between biofilm and antimicrobial resistance were limited in *Campylobacter*, positive impact of biofilm on reducing the permeation of ampicillin has been reported in other bacteria [49]. Some regulators, such as LuxS, have also been reported to be linked to biofilm formation and antimicrobial resistance in some bacteria [50]. We found an exception that the resistance rate to tetracycline was higher in non-biofilm producing isolates than in biofilm producing strains, it may be due to the high distribution of the resistance genes in non-biofilm producing isolates, such as *tet* [51]. It is interesting that all of the soil isolates and aerosol isolates were biofilm producers, which suggested that biofilm might be an important factor to help strain to survive in the surroundings as well as in the host. Our study on the biofilm-forming characteristics of *Campylobacter* isolates would help us understand the increasing resistance to antibiotics of *Campylobacter* as well as their pathogenicity to host.

In clade 3 and clade 4, 75% of the strains (20/25) were non-biofilm producers and the biofilm-forming abilities in these two clades were significantly lower than other clades (*p* < 0.05). The closely related strains may have a common ancestor, and STs developing from one biofilm-forming ST origin may share better biofilm-forming ability. The correlation of the origin and phylogenetic relationship between their *C. jejuni* isolates and biofilm-forming abilities has also been reported [52]. Previous studies also showed that some gene variants were associated with different *C. jejuni* multilocus sequence types, such as *fspA* [53] and *capA* [54]. The association between biofilm related genes and multilocus sequence types needs to be further studied. However, isolates within the same clade also exhibited varied abilities to form biofilm in our study. Ben et al. analyzed the genome sequences of strains with different biofilm-forming abilities, and found that three genes were associated with the increased biofilm formation in CC-21 and 43 genes in CC-45, but there was no overlap between these two CCs [55]. These results suggested a complex genetic correlation between genetic background and biofilm formation.

## Conclusions

In the present study, a high prevalence and genotypic diversity were observed in the *Campylobacter* strains isolated in chicken in central China. We analyzed the correlation among biofilm-forming abilities, MLST genotype and antimicrobial resistance, which revealed a positive correlation between resistance rate and the ability of biofilm-forming. This study will help us better understand the epidemiology and resistance of *Campylobacter*.

## Additional file

**Additional file 1: Table S1.** The details of each *Campylobacter* isolates.

## Abbreviations

MLST: multilocus sequence typing
STs: sequence types
CCs: clonal complexes
